# A Sex Pheromone Receptor in the Hessian Fly *Mayetiola destructor* (Diptera, Cecidomyiidae)

**DOI:** 10.3389/fncel.2016.00212

**Published:** 2016-09-07

**Authors:** Martin N. Andersson, Jacob A. Corcoran, Dan-Dan Zhang, Ylva Hillbur, Richard D. Newcomb, Christer Löfstedt

**Affiliations:** ^1^Department of Biology, Lund UniversityLund, Sweden; ^2^International Institute of Tropical AgricultureIbadan, Nigeria; ^3^The New Zealand Institute for Plant and Food Research LtdAuckland, New Zealand

**Keywords:** deorphanization, functional characterization, heterologous expression, HEK293 cells, odorant receptor, pheromone receptor, sensory neuron membrane protein

## Abstract

The Hessian fly, *Mayetiola destructor* Say (Diptera, Cecidomyiidae), is a pest of wheat and belongs to a group of gall-inducing herbivores. This species has a unique life history and several ecological features that differentiate it from other Diptera such as *Drosophila melanogaster* and blood-feeding mosquitoes. These features include a short, non-feeding adult life stage (1–2 days) and the use of a long-range sex pheromone produced and released by adult females. Sex pheromones are detected by members of the odorant receptor (OR) family within the Lepidoptera, but no receptors for similar long-range sex pheromones have been characterized from the Diptera. Previously, 122 OR genes have been annotated from the Hessian fly genome, with many of them showing sex-biased expression in the antennae. Here we have expressed, in HEK293 cells, five MdesORs that display male-biased expression in antennae, and we have identified MdesOR115 as a Hessian fly sex pheromone receptor. MdesOR115 responds primarily to the sex pheromone component (2*S*,8*E*,10*E*)-8,10-tridecadien-2-yl acetate, and secondarily to the corresponding *Z*,*E*-isomer. Certain sensory neuron membrane proteins (i.e., SNMP1) are important for responses of pheromone receptors in flies and moths. The Hessian fly genome is unusual in that it encodes six SNMP1 paralogs, of which five are expressed in antennae. We co-expressed each of the five antennal SNMP1 paralogs together with each of the five candidate sex pheromone receptors from the Hessian fly and found that they do not influence the response of MdesOR115, nor do they confer responsiveness in any of the non-responsive ORs to any of the sex pheromone components identified to date in the Hessian fly. Using Western blots, we detected protein expression of MdesOrco, all MdesSNMPs, and all MdesORs except for MdesOR113, potentially explaining the lack of response from this OR. In conclusion, we report the first functional characterization of an OR from the Cecidomyiidae, extending the role of ORs as long-range sex pheromone detectors from the Lepidoptera into the Diptera.

## Introduction

Insects are often dependent on communication via sex pheromones to reproduce. In many species of several insect orders, the sex pheromone is produced by the female, and detected by the male via specialized odorant receptors (ORs) that are located in the dendritic membrane of olfactory sensory neurons (OSNs) in the antennae (Wyatt, 2014). Insect ORs are members of a large and divergent family of 7-transmembrane proteins that are unrelated to the ORs of vertebrates (Kaupp, 2010). These ORs form ligand-gated ion channels of unknown stoichiometry together with a conserved co-receptor (Orco), although metabotropic signaling also seems to contribute to OR responses (reviewed in Carraher et al., 2015). OSN responses to pheromone components may also require the presence of certain sensory neuron membrane proteins (SNMPs), which are integral membrane proteins related to scavenger proteins of the CD36 family (Benton et al., 2007; Jin et al., 2008; Nichols and Vogt, 2008). SNMP1 affects responses of pheromone receptors in *Drosophila melanogaster* and moths, but its role in pheromone reception at the molecular level is just beginning to be unraveled (Benton et al., 2007; Li et al., 2014; Pregitzer et al., 2014; Gomez-Diaz et al., 2016). Rapid advances in next-generation sequencing techniques have resulted in an accelerating number of insect species for which sequences of ORs and other proteins involved in chemoreception have been identified (Montagné et al., 2015). Phylogenetic analyses of these receptors have revealed that most species possess one or several species-specific lineage expansions, while other lineages have been reduced or are simply no longer present in different insect groups (Nei et al., 2008; Ramdya and Benton, 2010; Hansson and Stensmyr, 2011; Benton, 2015). Amino acid sequence similarity has proven inadequate for inferring ligand specificity, therefore functional studies in heterologous systems have been employed to deorphan these receptors and address questions of how receptor function evolves across divergent insect taxa, how ecological specialization relates to OR specificities, and which molecular characteristics of ORs determine ligand selectivity (reviewed in Andersson et al., 2015). Functions of ORs have been studied most extensively among the receptors in the conserved sex pheromone receptor (PR) clade of Lepidoptera (reviewed in Zhang and Löfstedt, 2015), and among the ORs of the two major model species of Diptera, the fly *D. melanogaster* and the malaria mosquito *Anopheles gambiae* (Hallem and Carlson, 2006; Carey et al., 2010; Wang et al., 2010; Mansourian and Stensmyr, 2015). However, to better understand the functional evolution of ORs and their role in different ecological contexts, deorphanization of ORs must extend beyond the traditional model species.

The Hessian fly, *Mayetiola destructor* Say, is a herbivorous gall midge belonging to the large family Cecidomyiidae (Diptera; Gagné, 1989), which contains many agricultural pests (Harris et al., 2003; Gagné, 2004). Plant-feeding flies within this family share several intriguing ecological and life-history traits distinct from those of *Drosophila* and mosquitoes. Characteristics include a short adult life-span of 1–2 days or less (Gagné, 1989), the use of species-specific long-range sex pheromones (Hall et al., 2012), and in general a very narrow host range (Harris et al., 2003). Adult cecidomyiids have reduced mouthparts and do not feed, although they occasionally might drink water or nectar (Gagné, 1989). Cecidomyiids have also evolved an intricate relationship with their host plants, in which they induce galls to provide a diet with superior nutritional value for the developing larvae (Harris et al., 2006; Zhu et al., 2008), a trait that is thought to have contributed to adaptive radiation of gall inducing insects (Rohfritsch, 1992; Stone and Schönrogge, 2003). Phylogenetically, the Cecidomyiidae family lies within the suborder Nematocera, but is still well separated from mosquitoes (also Nematocera), and distinct from *Drosophila*, which resides within the suborder Brachycera (Yeates and Wiegmann, 1999; Lambkin et al., 2013).

The Hessian fly is a serious pest of wheat (*Triticum* spp.; Bouhssini et al., 1999; Berzonsky et al., 2003; Harris et al., 2003; Gagné, 2004). Like other cecidomyiids, its ecology is distinct from mosquitoes and other flies (Harris et al., 2003). All reproductive activities have to take place during a very limited time period (1–2 days) and the behavioral repertoire of adult *M. destructor* is limited. Females emerge with a full complement of mature eggs, start to release sex pheromone, and after mating search for host plants and oviposit until death occurs. Adult males more or less solely fly to calling females to mate, with no evidence for behavioral responses to plant odors (Foster et al., 1991a; Harris et al., 1993; Harris and Foster, 1999; Andersson et al., 2009).

Unlike *Drosophila* and mosquitoes, the female-produced sex pheromone of the Hessian fly is a multi-component blend that specifically attracts males over long distances, akin to the long-range attracting sex pheromone systems within the Lepidoptera (McKay and Hatchett, 1984; Foster et al., 1991a,b; Andersson et al., 2009). The female pheromone gland contains seven compounds that are active on the male antennae (Andersson et al., 2009). Six of the compounds have been identified, and a mixture of five of them is sufficient to attract males in the field (Andersson et al., 2009). While the synthetic sex pheromone mixture is useful for monitoring Hessian fly populations in the field (Andersson et al., 2009; Anderson et al., 2012; Schwarting et al., 2015), the identification of pheromone receptors from this species could be useful for future receptor-targeted control, for providing insights into pheromone detection at the receptor level, and amassing functional data for ORs in a large and intriguing group of Diptera.

The Hessian fly genome has been sequenced recently, and 122 genes predicted to encode ORs have been annotated, including a gene for Orco (Zhao et al., 2015). Phylogenetic analysis of these ORs, together with the ORs of *D. melanogaster* and *A. gambiae*, indicated that the majority (105 ORs) of the Hessian fly ORs form two major lineage-specific expansions, that entirely lack ORs from *D. melanogaster* and *A. gambiae* (Zhao et al., 2015). Analysis of expression levels in male and female antennae revealed that a strikingly large number of OR genes exhibit sex-biased expression, at least at the RNA level, with 50 genes being more highly expressed in females, and 12 more highly expressed in males, consistent with the observed sex-specific behavioral differences (Andersson et al., 2014). The majority of ORs with male-biased expression reside in a clade formed by MdesOR111-121, suggesting that the sex pheromone receptors might be found within this group. In addition, the Hessian fly genome is unusual in that it contains seven genes for SNMPs, of which six are closely related to SNMP1 and the seventh is a SNMP2 ortholog, with some members showing male- (SNMP1B and 1F) or female-biased (SNMP1A) antennal expression, and one member (SNMP1D) showing no, or very low, expression in the antennae (Andersson et al., 2014). At least three different, but non-mutually exclusive, effects of SNMP1 on pheromone reception have been suggested based on functional data. SNMP1 was shown to be necessary for responses of DmelOr67d to the pheromone *cis*-vaccenyl acetate (cVA) when analyzed *in vivo* in *Snmp*-mutated *D. melanogaster* (Benton et al., 2007), whereas another study indicated that SNMP1 affects the response kinetics, particularly the response offset, rather than being necessary for the initial response (Li et al., 2014). This effect was demonstrated for both the DmelOr67d receptor for cVA *in vivo*, and the *Bombyx mori* (Lepidoptera) PR for bombykol using *Xenopus* oocytes (Li et al., 2014). Also, SNMP1 has been shown to increase the sensitivity of a PR (HR13) from *Heliothis virescens* (Lepidoptera) in Human Embryonic Kidney (HEK) 293 cells (Pregitzer et al., 2014).

The aim of the present study was to identify the sex pheromone receptors of the Hessian fly and examine their requirement for SNMPs. Thus, we expressed five ORs residing in the above-mentioned clade of receptors with male-biased expression with or without each of the five antennally expressed SNMP1 genes in HEK293 cells, and tested the resulting cell lines for responses to all sex pheromone components identified to date in the Hessian fly. Here, we report the identification of the first sex pheromone receptor in the Hessian fly, which is also the first receptor for a female-produced long-range sex pheromone in a dipteran.

## Materials and Methods

### MdesOR and SNMP Cloning

Hessian flies, biotype Great Plains, were reared according to previously described methods (Harris et al., 2012; Andersson et al., 2014), and kindly provided by Prof. Marion O. Harris (North Dakota State University, Fargo, ND, USA). The insects used here were from the same strain previously employed for both genome (Zhao et al., 2015) and transcriptome (Andersson et al., 2014) sequencing. Briefly, cDNA libraries were made using the ThermoScript RT-PCR system (Thermo Fisher Scientific, Carlsbad, CA, USA), from RNA extracted from male or female antennae (from 122 and 145 individuals, respectively) using an RNeasy Mini Kit (Qiagen, Hilden, Germany) according to the manufacturer’s instructions.

We focused the study on ORs residing in the clade formed by MdesOR111-121 (Zhao et al., 2015), because we assumed that, similar to the receptors in the PR clade of Lepidoptera, the Hessian fly receptors detecting structurally similar pheromone components would be evolutionarily related to each other and have high expression, especially in the male antennae (Andersson et al., 2014). However, MdesOR111 was not included because of its very low expression levels in the antennae of both males and females, and MdesOR114 was dropped because this gene is not assembled into full-length in the current genome assembly (Zhao et al., 2015), and its complete open reading frame could also not be recovered from previous transcriptome data (Andersson et al., 2014). Furthermore, sequences of MdesOR117-119 and 121 could not be satisfactorily amplified from cDNA, with PCR products displaying premature stop codons, entire exons missing, and/or high sequence variation that made it impossible to accurately match them to any of the four gene sequences predicted from the genome assembly. Furthermore, *de novo* assemblies of the transcriptomic reads analyzed in Andersson et al. (2014) failed to verify the genomic sequences of these four genes. Hence, only the five remaining ORs in this clade (MdesOR112, 113, 115, 116, and 120) were included in the experiments. Cloning of the five ORs and the five antennally expressed SNMP1s largely followed the protocol described by Corcoran et al. (2014), starting either from male antennal cDNA (MdesOR112, 113, 120, SNMP1B, 1C, 1E, and 1F) or female antennal cDNA (MdesSNMP1A), or to fast track the project, from synthesized versions of genes (MdesOrco, OR115, and 116; purchased from GeneArt, Thermo Fisher Scientific) with gene sequences derived from the current genome and transcriptome assemblies (predicted sequences were identical in the two assemblies; Andersson et al., 2014; Zhao et al., 2015). Sequences of the synthetic genes were later cloned and verified from antennal cDNA, with all three genes sharing 100% predicted amino acid identity with those predicted from the genome assembly. Briefly, genes were amplified from cDNA using *PfuUltra II* Fusion HS DNA Polymerase (Agilent Technologies Inc., Santa Clara, CA, USA), and adenine residues were added to the PCR products through a final incubation with DreamTaq DNA Polymerase (Thermo Fisher Scientific). Products were then analyzed by agarose gel electrophoresis and purified using Wizard SV Gel and PCR Clean-Up System (Promega, Madison, WI, USA), following the manufacturer’s instructions. DNA fragments were then ligated into pTZ57R/T (Fermentas AB, Sweden) or pCR^®^8/GW/TOPO^®^ (Thermo Fisher Scientific; only MdesSNMP1F) vectors, and transformed into TOP10 competent cells (NEB, Ipswich, MA, USA). Successfully transformed clones were identified by PCR. Positive colonies were grown overnight in LB media, plasmids were isolated using the GeneJET Plasmid Miniprep kit (Thermo Fisher Scientific), and plasmids containing the correct sequence of target genes were identified by Sanger sequencing (Department of Biology, Lund University). For several of the genes, we observed single nucleotide polymorphisms (SNPs) when comparing their cDNA-derived sequence with their predicted genomic sequence. These SNPs were confirmed by sequencing multiple clones derived from at least two independent PCRs (to rule out PCR induced mutations), and clones showing the least number of SNP differences from genomic sequence were chosen for functional assays.

A second set of primer pairs was then used to add Kozak sequences, 5′ and 3′ restriction sites, and an N-terminal c-Myc (MdesOrco), N-terminal V5 (MdesORs) or C-terminal V5 tag (MdesSNMP1s) to genes of interest. Modified DNA was then digested with NotI/ApaI restriction enzymes, purified and ligated into pcDNA^TM^4/TO (MdesOrco), pcDNA^TM^5/TO (MdesORs) or pTREx-DEST30 (MdesSNMP1s) expression vectors (all Thermo Fisher Scientific) following the manufacturer’s protocols. Ligation products were transformed into competent cells, and positive colonies identified by colony PCR using full-length gene-specific primers as described above. Positive colonies were grown in LB media overnight, and large quantities of purified plasmids obtained using the PureLink^TM^ HiPure Plasmid Filter Midiprep Kit (Thermo Fisher Scientific) following the manufacturer’s instructions.

### Generation of an Inducible Isogenic Cell Line Expressing MdesOrco

HEK293 cell lines stably expressing MdesOrco, MdesORs and MdesSNMPs were produced and cultured according to previously described methods (Corcoran et al., 2014). All newly produced cell lines were passaged three times, frozen and stored at -80°C, and thawed prior to use in fluorescent calcium assays or additional transfections. Initially, an isogenic, tetracycline repressor-expressing (TREx) cell line was transfected with MdesOrco, and after confirmation of stable, inducible MdesOrco expression, the cell line (i.e., HEK293/TMO) was cultured in the presence of zeocin and blasticidin selection antibiotics (Gold Biotech), single-cell sorted (Corcoran et al., 2014), and the isogenic cell line that showed the most favorable growth rate and cell morphology was frozen and used for further transfection with pcDNA^TM^5/TO/MdesORs.

### Generation of Inducible Cell Lines Expressing MdesOrco, MdesORs, and MdesSNMPs

The isogenic HEK293/TMO cell line was used in five separate transfections with pcDNA^TM^5/TO/MdesORs (i.e., OR112, 113, 115, 116, and 120). Transfection conditions were identical to those for pcDNA^TM^4/TO/MdesOrco (Corcoran et al., 2014) except that FspI (New England Biolabs) was used for linearization of pcDNA^TM^5/TO/MdesOR113 and pcDNA^TM^5/TO/MdesOR116, and stable cell lines generated using 200 μg/ml hygromycin selection antibiotics (Gold Biotech) for approximately 2 weeks, after which the concentration was reduced to 100 μg/ml hygromycin and 200 μg/ml zeocin and 10 μg/ml blasticidin were added to the cell culture medium. Each of the five HEK293/TMO/MdesOR cell lines was used in five separate transfections with pTREx-DEST30/MdesSNMPs (i.e., MdesSNMP1A, 1B, 1C, 1E, and 1F). Transfection conditions were the same as those described above except that AhdI (New England Biolabs) and PciI (only SNMP1B) were used for plasmid linearization, and stable cell lines generated using G418 (Geneticin) antibiotics (Gold Biotech) at 625 μg/ml for approximately 2 weeks, after which the concentration was reduced to 250 μg/ml, and hygromycin (100 μg/ml), zeocin (200 μg/ml), and blasticidin (10 μg/ml) were added.

### Fluorescent Calcium Assays

In total, five HEK293/TMO/MdesOR cell lines, 25 different HEK293/TMO/MdesOR/SNMP1 cell lines, and one cell line only expressing MdesOR115 (HEK293/MdesOR115) were tested for responses to Hessian fly sex pheromone components in the previously described fluorescent calcium assay (Corcoran et al., 2014), as it is well established that insect OR complexes transport calcium upon ligand-induced receptor activation (e.g., Wicher et al., 2008; Pask et al., 2011). Briefly, cells were plated into 96-well plates, induced to express MdesOrco, MdesORs and MdesSNMPs, loaded with a calcium-sensitive fluorophore, and monitored for ligand-induced receptor activation using a FLUOstar Omega plate reader (BMG Labtech, Ortenberg, Germany).

Each cell line was tested for responses to the six known Hessian sex pheromone components (Andersson et al., 2009). The compounds, (2*S*)-tridec-2-yl acetate [2*S*-13:OAc, purity >99%], (2*S*,10*E*)-10-tridecen-2-yl acetate [2*S*-10*E*-13:OAc, >99%], (2*S*,10*Z*)-10-tridecen-2-yl acetate [2*S*-10*Z*-13:OAc, 66%], (2*S*,10*E*)-10-tridecen-2-ol [2*S*-10*E*-13:OH, 99%], (2*S*,8*Z*,10*E*)-8,10-tridecadien-2-yl acetate [2*S*-8*Z*,10*E*-13:OAc, 95%], and (2*S*,8*E*,10*E*)-8,10-tridecadien-2-yl acetate [2*S*-8*E*,10*E*-13:OAc, 99%] were purchased from PheroNet AB (Lund, Sweden), and purities analyzed by GC-MS. Regarding the compounds with conjugated double bond systems, the stereoisomeric purity of 2*S*-8*Z*,10*E*-13:OAc was 88%, containing 12% of its stereoisomers including 9% of the *E*,*E*-isomer, while that of 2*S*-8*E*,10*E*-13:OAc was 93%, containing 7% of its stereoisomers, including 2% of the *Z*,*E*-isomer.

Compounds were diluted in DMSO and assay buffer according to previously described methods (Corcoran et al., 2014). All sex pheromone components were initially screened for receptor activity at a concentration of 10 μM with 0.5% DMSO. Because several cell lines did not respond to any of the sex pheromone components at this dose, they were also screened at 30 μM stimulus concentration. In all screening experiments, each compound was loaded to each of three wells containing non-induced cells, and each of three wells containing induced cells (i.e., with expression of exogenous chemosensory genes turned on). Thus, each plate (biological replicate) included three technical replicates. Compounds identified as active (see below) on any of the OR or OR/SNMP cell lines were subsequently assayed in dose-response experiments. In these experiments, compounds were only tested on induced cells, and at threefold serial dilutions from 150 μM down to 7.6 nM with 0.5% DMSO. For all experiments, cells were only tested once, then discarded. A negative control (0.5% DMSO in assay buffer) was included in all experiments, and the Orco agonist VUAA1 was also tested on all 30 cell lines as well as the isogenic HEK293/TMO cell line expressing Orco in the absence of ORs and SNMP1s. VUAA1 was included to serve as a positive control for Orco expression because it has been shown to activate Orco in all insect species tested to date, including Orco proteins from several insect orders expressed in our HEK293 cell system (Corcoran et al., 2014; Corcoran et al., unpublished data). Wells of cells were tested once with a single compound or dose and discarded. For subsequent experiments (biological replicates), each cell line was re-plated from cell cultures, induced (or non-induced) and tested for response to compound. Responses to compounds were measured from at least three independent experiments.

### Western Blot and RT-PCR

Western blots were carried out to investigate whether proteins of MdesOrco, ORs, and SNMPs were present in the induced cells. Cells were cultured, induced, and pelleted as previously described (Corcoran et al., 2014). Corresponding non-induced cells were included as controls. Cell pellets were re-suspended in 200 μl lysis buffer containing 1X PBS, 2% DDM detergent (Glycon Biochemicals GmbH), and 1X protease inhibitor cocktail (Roche, Basel, Switzerland), and incubated at 4°C for 1.5 h. Tubes were occasionally inverted to mix the contents. Samples were then spun at 21,000 *g* for 30 min at 4°C, and the supernatants harvested. Total protein content was quantified using the BioRad DC Protein Assay kit and the above-described plate reader according to the manufacturer’s instructions. Twenty micrograms of total protein from each sample was mixed with 5X loading solution (50 mM Tris pH 6.8, 50% glycerol, 2% SDS, 5% 2-mercaptoethanol and 0.125% bromophenol blue) and incubated at 37°C for 30 min to denature proteins before loading onto a 4–15% Criterion^TM^ TGX^TM^ Precast gel (BioRad). Western blot analysis was performed by transferring the proteins onto a PVDF membrane using a BioRad Trans Blot Turbo, then blocking the membrane with 5% non-fat milk powder in TBST buffer (50 mM Tris-Cl, 150 mM NaCl, 0.05% Tween 20, pH 7.5), and incubating with a primary antibody [rabbit anti-myc antibody for myc-tagged MdesOrco; rabbit anti-V5 antibody (Cell Signaling) for MdesORs and MdesSNMPs, and an HRP conjugated secondary antibody (Cell Signaling)]. Epitope-containing bands were developed using the Pierce ECL Western blotting substrate and imaged with a BioRad ChemiDoc imaging system.

RT-PCR was performed to verify gene expression of ORs for one cell line in which the receptor protein was not detected by Western blot (MdesOR113). cDNA libraries were constructed with the ThermoScript RT-PCR system (Thermo Fisher Scientific) using Trizol-extracted RNA from both non-induced and induced pelleted cells as template (for details see Corcoran et al., 2014). Duplicate reactions were performed for each sample, with and without reverse transcriptase, to verify DNA elimination through DNase treatment prior to cDNA synthesis. Full-length gene specific primers were used in the PCR, and the product was cloned and its sequence verified by Sanger sequencing as described above. Additionally, it was recently shown in *Drosophila* that mammalian CD36 can substitute for SNMP1 (Gomez-Diaz et al., 2016); therefore, we also investigated whether the *Homo sapiens* CD36 (HsapCD36) homolog was expressed in the HEK293 cells, using full length gene-specific primers and the same cDNA and experimental procedure as for MdesOR113.

### Data Handling and Statistics

Except for the analyses of the response offset where all readings throughout the 1 min recording were included, only the first reading (i.e., 10 s post treatment) was used as a measure of response. In the vast majority of cases, the responses were strongest at 10 s post treatment, after which they gradually declined over time. Responses were analyzed and graphed in GraphPad Prism 6 (GraphPad Software Inc. La Jolla, CA, USA), where mean responses (±SEM) were calculated, and half maximal effective concentrations (EC_50_ and 95% CI) from dose-response assays estimated using the non-linear curve fit regression function. Only ligands that elicited significantly stronger responses in induced compared with non-induced cells in the screening experiments were regarded as active. Thus, to identify active ligands, general linear models with “induction (yes/no)” as a fixed factor and “plate (#1, 2, or 3)” as a random factor to account for inter-plate variation, were performed for each sex pheromone compound and each cell line independently. Within each cell line, responses in induced cells to different active compounds were subsequently analyzed using a similar model, but with “compound” as a fixed factor, and again “plate” as a random factor. In both of these analyses, Δ-fluorescence [%] 10 s post loading was used as a dependent variable. Analysis of covariance (ANCOVA) was performed to investigate whether the presence of SNMP had any effect on the response offset. In these models, “SNMP” (no SNMP, or SNMP1A, B, C, E, or F) was included as a fixed factor and “time post treatment” as a covariate. All statistical analyses were performed in IBM SPSS Statistics v 22.

## Results

We generated 30 cell lines transfected with MdesOrco in combination with each of the five MdesOR genes with male-biased mRNA expression (MdesORs 112, 113, 115, 116 and 120), either on their own or in combination with one of the five antennally expressed MdesSNMP1 genes (SNMP1A, B, C, E, or F). DNA sequences of the cloned chemosensory genes as contained within their respective expression vector, including the added epitope tags, Kozak sequence and restriction sites are presented in Supplementary Data Sheet 1, along with highlighted synonymous and non-synonymous polymorphisms (the latter ranging from 0 to 7, median = 1) in comparison to their sequences predicted from the genome assembly. The cDNA sequences have been submitted to GenBank (accession numbers: KX661029–KX661039).

**FIGURE 1:**
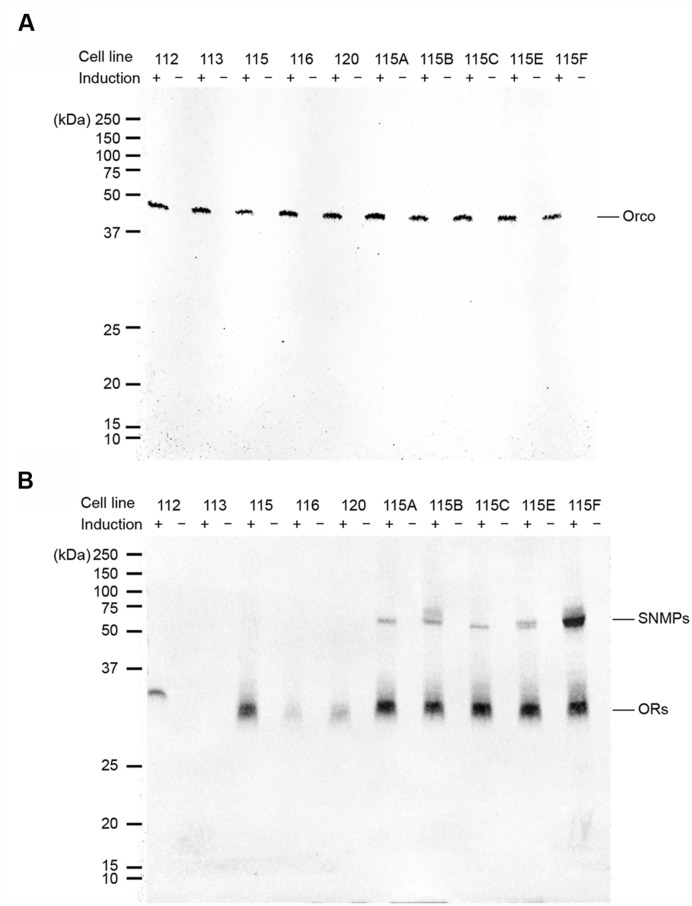
**Protein expression of myc-tagged Orco (A) and V5-tagged ORs and SNMPs (B) in cell lines with induced (+) gene expression, as indicated by Western blots.** No proteins were detected from non-induced cells (-). Numbers (112, 113, 115, 116, and 120) refer to the MdesORs, and letters (A, B, C, E and F) to the SNMP1 paralogs. The OR113 protein could not be detected.

Western blots were performed to detect proteins of MdesOrco, the ORs and SNMPs among selected cell lines. These proteins were never detected from non-induced cells (**Figure 1**). Myc-tagged Orco was detected from all induced cell lines that were assessed (**Figure 1A**). Proteins of V5-tagged MdesOR112, 115, 116, and 120 were also detected, however, OR113 was not (**Figure 1B**), also after repeated blotting. All V5-tagged SNMP1 proteins were detected from the different MdesOrco/OR115/SNMP1 cell lines. SNMP1B, and to some extent 1E, was detected as a double band. Apart from Orco for which cell lines were isogenic, the other proteins were detected at varying band intensities, which may reflect differences in levels of protein present in different cell lines. Using RT-PCR, full-length MdesOR113 was amplified from induced cells and not from non-induced cells, suggesting that MdesOR113 is expressed at least at a transcriptional level, and that its expression is being properly regulated by the TREx system. We also amplified and verified the sequence of HsapCD36 in both induced and non-induced cells, suggesting that HsapCD36 is endogenously expressed by the HEK293 cells and is not regulated by the TREx system. Targets were not amplified from (-)-reverse transcriptase controls, verifying that PCR products reflected MdesOR113 and HsapCD36 mRNA expression in cells.

**FIGURE 2:**
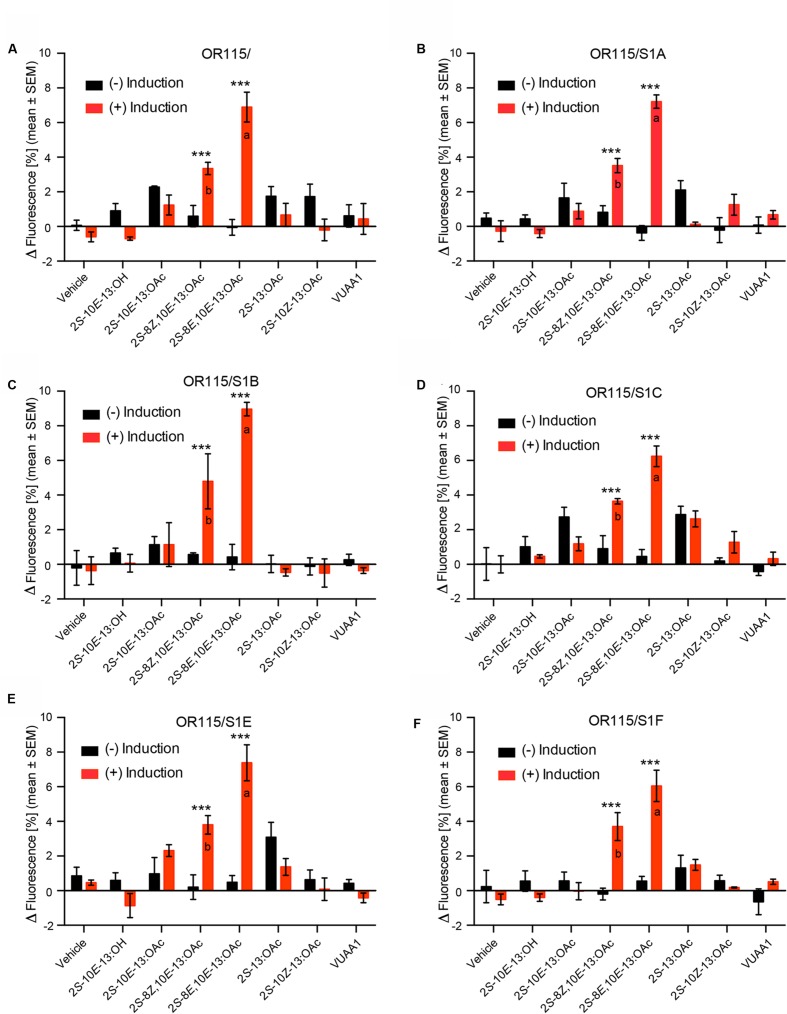
**Responses of six HEK293/TMO/MdesOR115 cell lines, with or without SNMP1 (S1), to Hessian fly sex pheromone components (10 μM concentration), including both induced cells (red bars) and non-induced cells (black bars).** Shown are also responses to vehicle control (0.5% DMSO) and the Orco agonist VUAA1 (50 μM). Asterisks indicate significant differences between non-induced and induced cells (all *p* < 0.001). Different lower-case letters indicate significant (all *p* < 0.007) differences between the response to 2*S*-8*Z*,10*E*-13:OAc and 2*S*-8*E*,10*E*-13:OAc. Means (±SEM) are derived from three biological replicates (*n* = 3), each including three technical replicates (i.e., total *n* = 9). Cell lines: **(A)** HEK293/TMO/MdesOR115, **(B)** HEK293/TMO/MdesOR115/SNMP1A, **(C)** HEK293/TMO/MdesOR115/SNMP1B, **(D)** HEK293/TMO/MdesOR115/SNMP1C, **(E)** HEK293/TMO/MdesOR115/SNMP1E, **(F)** HEK293/TMO/MdesOR115/SNMP1F.

All of the above-mentioned 30 cell lines were challenged with the six pheromone compounds in fluorescent calcium assays. Our screening trials using a stimulus concentration of 10 μM revealed that the HEK293/TMO/MdesOR115 cell line devoid of any SNMP1 responded to Hessian fly sex pheromone components (**Figure 2A**). Both of the two double-unsaturated acetates 2*S*-8*E*,10*E*-13:OAc and 2*S*-8*Z*,10*E*-13:OAc elicited responses in the HEK293/TMO/MdesOR115 cell line, with responses to these two compounds being significantly stronger in the induced compared to the non-induced cells (ANOVA, *E*,*E*: *F*_1_,_14_ = 123.9, *p* < 0.001; *Z*,*E*: *F*_1_,_14_ = 21.1, *p* < 0.001). The response to the *E*,*E*-isomer was stronger than the response to the *Z*,*E*-isomer (*F*_1_,_14_ = 33.8, *p* < 0.001). All of the HEK293/TMO/MdesOR115 cell lines that also contained an MdesSNMP1 protein showed similar responses as the MdesOR115 cell line without SNMP1, with 2*S*-8*E*,10*E*-13:OAc and 2*S*-8*Z*,10*E*-13:OAc eliciting significantly stronger responses in induced cells than in non-induced cells (*E*,*E*: *F*_1_,_14_ = 52.0–126.2, all *p* < 0.001; *Z*,*E*: *F_1_,_14_* = 18.1–35.0, all *p* < 0.001). In these five cell lines, the *E*,*E*-isomer also elicited stronger responses than the *Z*,*E*-isomer (*F*_1_,_14_ = 10.1–23.8, all *p* < 0.007; **Figures 2B–F**). As expected, the HEK293/MdesOR115 cell line (devoid of both Orco and SNMP1) did not respond to compounds, suggesting that Orco is necessary for pheromone responses of MdesOR115. The vehicle control (0.5% DMSO) did not elicit a response in any of the cell lines included in the study. Also, the Orco agonist VUAA1 did not elicit a significant response in any of the cell lines, even though Orco was detected by Western blotting. The isogenic HEK293/TMO cell line expressing MdesOrco in the absence of ORs and SNMP1s also did not respond to VUAA1.

**FIGURE 3:**
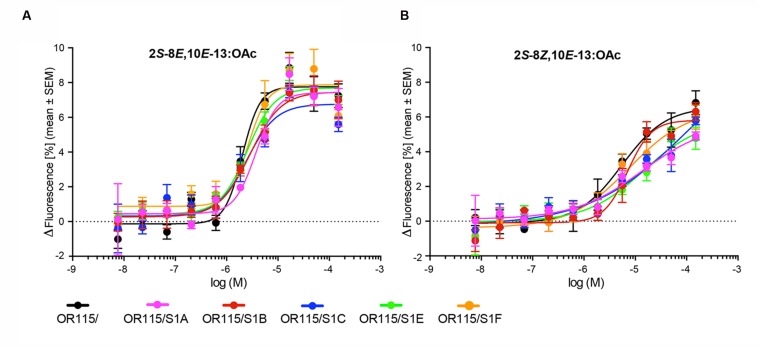
**Dose-response curves of six HEK293/TMO/MdesOR115 cell lines, with or without SNMP1 (S1).** Means (±SEM) are derived from three to four biological replicates (*n* = 3–4), each including three technical replicates (i.e., total *n* = 9–12). **(A)** Responses to 2*S*-8*E*,10*E*-13:OAc, and **(B)** 2*S*-8*Z*,10*E*-13:OAc.

The six responsive HEK293/TMO/MdesOR115 cell lines (with and without any SNMP1) were all subjected to dose-response assays using 2*S*-8*E*,10*E*-13:OAc and 2*S*-8*Z*,10*E*-13:OAc as stimuli. These assays indicated very similar dose-response curves for all six cell lines, with responses to the *E*,*E*-isomer starting to appear at the 0.6 μM concentration, and maximal responses reached at the 17 μM concentration (**Figure 3A**). The EC_50_ values were also similar among the six cell lines with all showing overlapping 95% CI. The average EC_50_ values estimated for the six HEK293/TMO/MdesOR115 cell lines ranged from 2.02 to 3.69 μM. Dose-response curves for the *Z*,*E*-isomer (**Figure 3B**) were also similar between the six cell lines. However, these curves were not sigmoidal, rendering estimations of EC_50_ values inaccurate. Taken together, we find no evidence to suggest that SNMP1 is necessary for response to sex pheromone components, nor does SNMP1 seem to affect the sensitivity or magnitude of the response of MdesOR115 to its active ligands in this *in vitro* system.

Cell lines transfected with any of the other four OR genes, with or without SNMP1 (i.e., 24 cell lines in total), did not respond to any of the pheromone components at the 10 μM or 30 μM concentration (data not shown).

**FIGURE 4:**
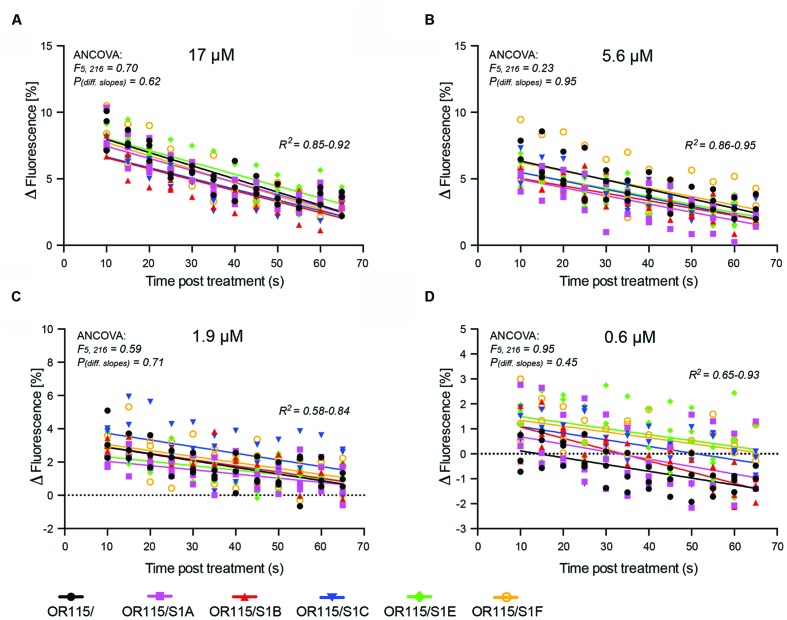
**Temporal response change post treatment of 2*S*-8*E*,10*E*-13:OAc in six HEK293/TMO/MdesOR115 cell lines, with or without SNMP1 (S1). (A–D)** show responses to decreasing ligand concentrations. Analyses of covariance show that the slopes do not differ between different cell lines. Each data point (biological replicate) is the average of three technical replicates (*n* = 3–4 biological replicates, total *n* = 9–12).

It has been suggested that SNMP1 affects the kinetics of pheromone responses, especially the response offset (Li et al., 2014). We addressed whether or not the five SNMP1 proteins affected the response offset by analyzing the response change over time starting from the first reading. We found no effect of SNMP1 on the rate of response change post stimulation across the tested concentration range (**Figure 4**; Supplementary Image 1).

## Discussion

The sex pheromone gland of the Hessian fly female contains seven compounds that activate the male antennae, six of them are chemically identified, and a blend of five components is sufficient to elicit strong attraction of males in the field (Andersson et al., 2009; Anderson et al., 2012; Schwarting et al., 2015). We here report the identification of MdesOR115 as the first receptor capable of detecting essential components of this sex pheromone blend. Although receptors for mating attractants and the male pheromone cVA have been identified previously in *Drosophila* (van der Goes van Naters and Carlson, 2007; Dweck et al., 2015), this receptor in the Hessian fly, to our knowledge, is the first to be described that detects components of a long-range female-produced sex pheromone within the Diptera. It is also the first functionally characterized OR from a species belonging to the Cecidomyiidae family, a large group of Diptera that is ecologically and phylogenetically distinct from the extensively studied model species *D. melanogaster* and *A. gambiae* (Hallem and Carlson, 2006; Carey et al., 2010; Wang et al., 2010; Mansourian and Stensmyr, 2015).

MdesOR115 responded to the two dienes of the sex pheromone blend, displaying preference for 2*S*-8*E*,10*E*-13:OAc over the corresponding *Z*,*E*-isomer. Both of these pheromone components are present in minute amounts in female gland extracts, but they elicit powerful responses in male antennae, and both are required for optimal male attraction (Andersson et al., 2009). However, the preference of OR115 for the *E*,*E*-isomer might be higher than that indicated by the data. Compounds with conjugated double bond systems are in general difficult to synthesize and store as pure stereoisomers and our purity analysis using GC-MS indicated that the *Z*,*E*-compound contained 9% of the *E*,*E*-isomer. It is therefore likely that part of the response by MdesOR115 to the *Z*,*E*-isomer was due to the presence of 2*S*-8*E*,10*E*-13:OAc in the stimulus, and therefore this receptor might be more specific for the *E*,*E*-isomer than it appears. This observation is consistent with previous electrophysiological recordings from single OSNs of the Hessian fly, with one class of neuron responding specifically to 2*S*-8*E*,10*E*-13:OAc and another to 2*S*-8*Z*,10*E*-13:OAc (Boddum et al., 2010), although an exact correspondence might not always be expected due to the presence of, for example, odorant binding proteins (OBPs) and odor degrading enzymes (ODEs) in the living insect (Leal, 2013). The dose-response curves for the *Z*,*E*-isomer were not sigmoidal. The most likely explanation for this observation is that the curves did not reach a plateau even though stimulus concentrations as high as 150 μM were tested. Higher concentrations were not tested because responses also in non-induced cells and compound solubility issues started to appear at such high concentrations. Nevertheless, this finding indicates that the *Z*,*E*-isomer is a rather poor ligand for MdesOR115, irrespective of whether or not part of the response was due to the presence of the *E*,*E*-isomer in the stimulus. The high selectivity of MdesOR115 in discriminating structurally similar sex pheromone components is in accordance with the response pattern of several PRs and pheromone OSNs in moths where only one or a few compounds are active. Saying this though, there are also examples of broadly tuned moth sex PRs (reviewed in Zhang and Löfstedt, 2015).

OSN responses in the Hessian fly have also been recorded to 2*S*-10*E*-13:OH, and 2*S*-10*E*-13:OAc, but not to 2*S*-13:OAc (2*S*-10*Z*-13:OAc has not been tested; Boddum et al., 2010). However, none of the tested Hessian fly ORs responded to these compounds. In fact, of the five tested ORs, all showing male-biased expression, four did not respond to the sex pheromone components. Three of the non-responsive ORs (MdesORs 112, 116 and 120) were detected by Western blot, indicating that they were present as proteins in the cells, although their band intensities were lower than that for OR115. The lack of response in these ORs suggests that they might be tuned to compounds not included in the test odor panel, such as the seventh unidentified compound present in female pheromone gland extracts (Andersson et al., 2009). Thus, future studies on these ORs should include a gland extract to investigate this possibility. Moreover, it is possible that the non-responsive receptors are translated in insufficient numbers or fail to incorporate properly with Orco in the HEK cell membrane, or that they otherwise malfunction in the employed heterologous system. An additional possibility is that the presence of the artificial epitope tag affects the response of the MdesORs, however, data from other species show that the tags do not influence the receptors’ pheromone response (Corcoran et al., 2014; Mansourian et al., 2016; Corcoran et al., unpublished data). On the other hand, although OR113 could be amplified from cDNA of its corresponding cell line, its protein was not detected, which might indicate that OR113 is not translated in HEK293 cells or that the cells produce a protein where the tag is cleaved or unavailable for detection by the antibody. Absence of odor responses and functional OR expression have been observed also in previous studies for several ORs when expressed in various heterologous systems (Hallem and Carlson, 2006; Carey et al., 2010; Wang et al., 2010). Similar to other studies (Pellegrino et al., 2011; Arguello et al., 2016), we observed SNPs among the cloned chemosensory genes, a finding that is expected for rapidly evolving genes when using RNA samples pooled from >100 individuals. Whether the observed SNPs affect the proteins’ function remains unknown, but since males of the present lab culture fly readily to females to mate, presumably attracted by their pheromone, it is likely that their pheromone detection system remains intact. Surprisingly, we also found that the Orco agonist VUAA1 did not elicit a response in MdesOrco, which to our knowledge is the first reported Orco that does not respond to this compound. Despite it being unresponsive to VUAA1, the fact that MdesOrco was required for MdesOR115 responsiveness to pheromone indicates its functionality in cell lines and crucial role in Hessian fly pheromone reception. We are currently investigating the molecular basis underlying the lack of MdesOrco responsiveness to VUAA1. Future studies on additional insect species are needed to investigate if the loss of VUAA1 response is unique to the Hessian fly Orco or common across the Cecidomyiidae family, or whether it also has occurred independently in additional insect taxa.

MdesOR115 belongs to a subfamily of ORs whose transcripts are present at higher levels in male compared to female antennae (Andersson et al., 2014). This subfamily, in turn, belongs to a larger receptor lineage that appears to have expanded in the Hessian fly, containing no receptors from *D. melanogaster* or *A. gambiae* (Zhao et al., 2015). In that dendrogram, this Hessian fly-specific lineage was sister to a group containing four ORs from *A. gambiae* (AgamORs 43, 44, 66 and 67; Zhao et al., 2015), all of which remain orphan. Thus, MdesOR115 is not closely related phylogenetically to any other OR of known function. Interestingly, however, is that of all the ORs encoded by the *D. melanogaster* genome, OR67d, which detects the contact pheromone cVA (van der Goes van Naters and Carlson, 2007), is the one DmelOR that is the most closely related to MdesOR115 (Zhao et al., 2015). Although its phylogenetic position is fairly well separated from that of MdesOR115, this observation makes it tempting to speculate that the detection of these sexual signals might have a single evolutionary origin. In contrast to DmelOR67d, the other two DmelORs that detect sexual signals in *Drosophila* (DmelOR47b and DmelOR88a; Dweck et al., 2015), are phylogenetically more distant from MdesOR115 in the dipteran OR phylogeny (Zhao et al., 2015). Among the ORs in the clade with male-biased transcript levels in the Hessian fly, MdesOR115 (and MdesOR112) has reasonably high expression in females as well, with mRNA levels comparable to several of the ORs outside of this clade that have female-biased expression (Andersson et al., 2014). Although mRNA levels do not necessarily reflect actual protein levels, this observation suggests that females might be able to detect the double-unsaturated compounds of their own pheromone. Similar observations at the mRNA level have been made among receptors in the conserved PR clade of moths, with some receptors even showing female-biased expression (Widmayer et al., 2009; Liu et al., 2013; Corcoran et al., 2015).

In addition to pheromone receptors, SNMP1, which shows widespread conservation among insects (Rogers et al., 1997; Nichols and Vogt, 2008; Andersson et al., 2013; Jiang et al., 2016), is important for pheromone reception in both *D. melanogaster* and moths (Benton et al., 2007; Li et al., 2014; Pregitzer et al., 2014). Three non-mutually exclusive effects of SNMP1 on pheromone reception have been observed, namely (i) that SNMP1 is required for pheromone response (Benton et al., 2007), (ii) that it provides a more rapid onset and especially offset of response (Li et al., 2014), or (iii) that it increases the sensitivity of pheromone detection (Pregitzer et al., 2014). However, none of these effects could be confirmed in our study for any of the five SNMP1 paralogs. This somewhat unexpected result might suggest that SNMP1 is not important for pheromone detection in the Hessian fly, or at least not for the detection of the two dienes by MdesOR115. It is, however, possible that the different SNMP1 paralogs interact with ORs or ligands not included in the present study. Alternatively, the lack of any observable effects of SNMP1 on the response of MdesOR115 could possibly be explained by the use of different *in vivo* and *in vitro* systems in the different studies, which all differ in their cellular, molecular, and chemical properties.

The mechanism by which SNMP1 interacts with other proteins involved in chemoreception is not fully understood but a putative model was recently proposed by Gomez-Diaz et al. (2016), supported by their experimental data on DmelSNMP1 and previously proposed models (Rogers et al., 2001; Benton et al., 2007; Li et al., 2014; Pregitzer et al., 2014). Similar to other members of the CD36 family that bind and transport lipids and lipoproteins (Ge and Elghetany, 2004; Martin et al., 2011), SNMP1 can bind lipophilic pheromone molecules (Gomez-Diaz et al., 2016). Structure-activity experiments demonstrated that the large ectodomain of SNMP1 is essential for pheromone (cVA)-evoked activity of OSNs in *D. melanogaster*, where it seems to form a tunnel that may transport lipophilic pheromones in the extracellular fluid to the membrane receptors (Gomez-Diaz et al., 2016). Based on the findings of Li et al. (2014), which suggest that SNMP1 facilitates both the association and disassociation between the pheromone ligand and its receptor, it is possible that the SNMP1 ectodomain is able to transport pheromones both to and from the ligand-binding pocket of ORs (Gomez-Diaz et al., 2016). However, it is unknown whether this proposed mechanism applies to all SNMP1s in species where multiple paralogs have evolved, such as the Hessian fly. It is possible that the different SNMP1 paralogs have different ligand affinities, or that some of them have acquired olfactory functions unrelated to pheromone reception or perhaps even non-olfactory functions. Due to the lack of effects of any of the SNMP1s tested in the present study, this hypothesis remains to be tested. Interestingly, however, Gomez-Diaz et al. (2016) also showed that *Mus musculus* CD36 partially restored the cVA response in *Snmp*-mutated *D. melanogaster*, and we show that our cell lines express the HsapCD36 gene. Whether HsapCD36 has the same effect on pheromone detection as the mouse homolog is unknown, but the expression of endogenous HsapCD36 in our cell lines could at least partly underlie the observed lack of effect of the MdesSNMP1 paralogs.

Li et al. (2014) showed effects of SNMP1 on pheromone response kinetics, with the strongest effect observed for receptor deactivation. We did not observe any trend that SNMP1 induces a faster return to baseline post treatment. A crucial difference, however, between our HEK293 cell system and the systems used by Li et al. (2014) is that the stimulus remains in the well in our HEK cell system, but is cleansed by wash or degradation (*in vivo*) in the other systems. In the absence of ODEs, the continuous presence of the stimulus in our HEK cell system could possibly obscure an effect of SNMP1 on the response offset, because the ligand might repeatedly associate and disassociate with the receptor. Irrespectively, based on current incomplete mechanistic knowledge of SNMP1 function, the large number of SNMP1 paralogs in the Hessian fly, and the various roles of CD36-related proteins, we can only speculate about possible technical causes underlying the observed lack of effects *in vitro* (except for protein expression being an issue), and conclude that possible roles of SNMP1 in pheromone detection in the Hessian fly remain elusive and should be further investigated.

## Author Contributions

MA, JC, YH, RN, and CL conceived, designed, and coordinated the study. MA performed molecular work, cell culturing, functional assays, and data analysis. JC performed cell culturing, and assisted in molecular work, functional assays, and data analysis. D-DZ assisted in molecular work. MA drafted the manuscript, with all co-authors providing editorial and scientific advice. All authors have read and approved the final version of the manuscript.

## Conflict of Interest Statement

The authors declare that the research was conducted in the absence of any commercial or financial relationships that could be construed as a potential conflict of interest.
