# Supplementary material for: A Sex Pheromone Receptor in the Hessian Fly Mayetiola destructor (Diptera, Cecidomyiidae)
Source: Front Cell Neurosci. 2016 Sep 7;10:212. doi: 10.3389/fncel.2016.00212 (PMC5013046; doi:10.3389/fncel.2016.00212)
Supplement: Supplementary file 1 [file Data_Sheet_1.DOCX]

**Supplementary Dataset 1.**

DNA sequences of MdesOrco, MdesORs, and MdesSNMP1s as they appeared in their respective expression vector. The added Kozak region, ATG start codon, and stop codon are underlined. Sequences corresponding to the added V5 or c-Myc epitope tags are in bold, and recognition sites for NotI and ApaI restriction enzymes in italics. Synonymous SNPs compared to the predicted genome sequence are highlighted in green, and non-synonymous SNPs in red, with corresponding amino acid changes (genome🡪cDNA) listed in order of appearance below each sequence.

>MdesOrco

*GCGGCCGC*CACCATG**GAGCAGAAGCTGATCTCCGAGGAGGACCTG**CAGGTCCAGCAGAAGCAATTCACAGGACTTGTGGCAGATTTAATGCCCAATATTCGGCTGATGCGTTTTCTTGGCCATTTTTTGCATAAGTTATCGAATGGGCCAACATTTCTCAGTAAATTATACTCGATGATGCATCTGATGTTAATGCTCTTACAATTTTTCTGTATCATCATTAACCTCGCATTGAATACAAGCGAAGTGAATGAATTAACCGCCAACACAATCACAACACTGCATTTCACACACACAATCACAAAGTTCTGTTATTTGGCGATCAACAACAAAAACTTCTATCGTACATTCAATATTTGGAATCAATCAAATTCTCATCCCTTGTTTGCTGAGTCTGATGCTCGCTATCATAGTATTGCGTTAGCTAAAATGCGCAAGAATCTTTATATGATCACCGGCTTAACAATGGGAACCGTTGCATCCTGGAGCATTATAACGTTCTTCGGTGAAAGCGTTAAAGGTGTGTTTGACAAAGAAACCAATGAAACGTACTATGTTGAGGTTCCGCGTTTGCCAATTAAATCTTACTATCCGTGGGATGCCATGTCTGGCATGAAATATATCGGAACATTCGTTTTCCAGGTTTATTATTTGACCTTCTCAATGCTGGCTTGTAATTTGACTGATGTCCTCTTCTGTTCGTGGCTATGTTTTGCCTGCGAGCAATTGTGTCATTTGAAGGGTATCTTAAAACCTCTTATGGAATTATCAGCATCATTGGACACATATCGGCCAAATTCAGCAGCGTTATTTCGCTCATTGTCTGCTAATTCAAAGTCTGAATTGGTTAATAATGATGAATATGATCAACAAGATGAAAATGGCAGCGTTTTTGTGCCAAAAGCTGAATGGGGTGGTACTTTCAAGGTTCCTCCAAACATGGGCAATCCAAATGGTTTGAGTAAAAAACAAGAAATCATGGTTCGTAGTGCAATTAAATATTGGGTTGAACGCCATAAGCATGTCGTAAGACTTGTTTCGGCCATCGGTGATACATATGGTGCTGCTCTTCTTTTACATATGTTGACTGCAACTATTTGCCTTACTCTGTTGGCATATCAAGCGACAAAGATCGATGGCGTTAACGTTTATGCCTTCACAACAATTGGATATCTTGTTTATTCACTTGGTCAAGTTTTCCATTTCTGTATTTTTGGGAATCGATTAATTGAGGAGAGTTCATCGGTGATGGAAGCAGCTTATTGTTGCCATTGGTACGACGGGTCAGAGGAAGCAAAGACTTTCGTCCAAATTGTTTGTCAACAATGTCAGAAAGCAATGACAATTTCAGGAGCCAAGTTCTTTACTGTGTCACTTGATTTGTTCGCATCCGTACTTGGCGCTGTAGTAACTTACTTCATGGTCTTAGTGCAACTTAAGTAA*GGGCCC*

>MdesOR112 sequence in pcDNA5TO

*GCGGCCGC*CACCATG**GGCAAGCCTATCCCTAATCCTCTGCTGGGCCTGGACAGCACC**AAAATTTTACATAAAATATCGAAATTAAAACAAGATTGGAATACCAGTTCACCGATATGGAGATTGAATCGTTTATTAAAGGTCGGTAAATTTGGTGGTTACTTAGTTGGGATCCGATTTCTTGGGGACTGTAAAATTGTTTGGTATTCGGGTAAAACTGGATTTGTTATCTCAATATATTATATATTGGCAATTTATACATTGATCCATTATGCTATG**G**AAAATCGAATTGTTGATGGATTACCCTGTTGGAGCATGAGCGGACTCTATCTTTCGTCCATGGTTGCTTATGCTAATGCATTAACAAAAAGACATCGTATCTATAAAATTGGACATTTTGGACAAACTTATTTGTACAACGAAGA**A**TTTGATGAAATTTGCTCGAAAAGTGTTAACAAAACTGTCAAAAG**A**TTTATGATAATAATGGGTATAATTCTTGCATCTGTGATACTTGGAAGCATTAATCCTGTTTATATTTGGTATCGTGATGGTATTTTCTACAGTTTAGCCGGTGTTCGATTTCCATTTGTCGAACAAAATAGCAATTTGAATATTATGTTAAATATTATCCTTCAATTGCTACTTGGATACAATGCAATAAATGGATTGACGTTGCTTCA**G**ACGTACCAGGGAACTCTTATGAATGCTATTGAATTGTCAGCCGACATTTCCATAAAAGAAATGATAAATCTATCCGATTATTTGGAAGGTGGTAATGCAGATGATGCC**G**TTATTAAAAAACATATCACAAAAATATTCAGACAAATCCAACGTATAGACGGTTATATTAAGAATGCATCAGATTGTCACTATTGGTACTTTTTTCTTTCATCCCCTTTTATCACATATTCGATTGGATTGTCGATCTATGCCCAGTACATTATGGGATTTCCTCCCGGATATGGAATAGCCCTCATGTCCTATGTTCAAATGGCTGTTTTGT**A**CTATATTGGTCAAGTCGTTGTTGACAGGAATAATCGCATCCGTTATAGTTTGTATTCGGACTTGAAATGGTATCTGTTACCACCCAAATACCAGCGAGATGTTTGTTTTATGATGAAACGTATGCAAAATGGGGCTACTCTCACTATTGGACCATTTCAAACTTTGAATTTCGAAACACTGAGAATTTTAACTCAAAGGATCCAATCTTTTATTATGTTTTTGTTAAATTTCCATTAA*GGGCCC*

Amino acid differences compared to genome: Q🡪E; I🡪V; C🡪Y

>MdesOR113 sequence in pcDNA5TO

*GCGGCCGC*CACCATG**GGCAAGCCTATCCCTAATCCTCTGCTGGGCCTGGACAGCACC**AAAGTGATCAAAGAACTGCGTGAAAAGTGGCATGGATGGCCAACATTTTGTCCAAAAAAGAAATGGCTTTTATTCTGTAATTTTGGTAAAAAAGTTAGCTATTTGGTTGGGCTCCGAGTTCAGGGCGATGCAAAAATTACTAGGATGACCTATTTCACTTTAGCCCTGGGAGGAGTTTATTACATTTTTGCTCCTTACACCATAATTATCTATGCATTAGAAGGCCGATTATCGGAAGGTTTTTCTTGTTTGAGTTTGAGTGGCCTATATACTTCGGCAGTGATAGCTCAAATTATATCAAGTTTTTCTGATGCTCGTTTCGATGCAAGGGATCTTTTTCAATTTGCAATTAAAAACATACACAA**C**AACGATCACGATGCAAATGAATATAATGCCATTTGCACAAAAAATATTCACATTACGATTCGACGATTTATTTCAGCGCTTATCTTACTAACTGCATCTTTTCTTGTTGGTGTTGCAAATCCCGTATATATTTTCATTAAAAATGGAAAATTTTATACTCTTGCTGGAATTT**T**GTTTCCATTTGTGGAAAAAGATTC**A**AAAACTGAGCTTTATATAAATATAATTTATAGCTTGGTAGTTATAATGGTCGCTG**T**CTTGGCTGTTATAGGCATACAAGTTTGCTGTGGAATTATATGTCA**T**ACCATAGAGGTGACGGCTGATTTGAGTATAAATGAAATGAAAGAATTATCCATCAATTTGGAAATGAACAAAACGAATGAAGCAAATATTGGCGTAAAGCTCAATCGAATATTTCAACAAATCCAGAAAATTGACGAATATATTATAAAGACTGGTAAAGCTTATTTTTGGTTTTTTTTCATTTCACCAATATTGTATACCTATTCGATTGGAGTGGCCATTTACAGTCAATGGATGAACGGATTTCCACCGGGTTACGG**G**ATAGGCGTAATCGGATATGTTCAAATGGTCATATTAGGATTAATGGGACAGATTAGTGTCGATCGTAACGAAAAACTAAGGGATGCTCTGTATTCGGAA**C**TAAAATGGTATCTACTACCAGTGAA**A**CGTCAGCGAGAT**G**TTTGTCATGTAATGAATCGATTACAAAATGGTGCCGTATTAACAATAGGACCGTTCGAAACGTTGAACTTTGAAGTTATTAAAATTTTAACGCAACGCATACACTCGTTTCTGATGCTTTTGATCAATTTTTCAAAATGA*GGGCCC*

Amino acid differences compared to genome: S🡪L; A🡪V; I🡪V

>MdesOR115 sequence in pcDNA5TO

*GCGGCCGC*CACCATG**GGCAAGCCTATCCCTAATCCTCTGCTGGGCCTGGACAGCACC**GAATTCACCAAAAGTCCTTTCAAAATTTGGTCGGTTGTGAAAGAAATGCGAAAAATTTACAATAATTGGGACGCACTCACTCCAGCCAAACAAATCTATTACATATACGAATTGGGCAGATTTTCTGGAATAGCGGCTGGTTTTCGTTCGATGCATGATTGTCGCATCGTTTTATCTTCATTTATGTTGGCCATCATTCTCCTAATGTATTACATTTTGGCATTTTACACGATCTATATTCGAACAATCGATGGTCGATTTGCGGAAGGTCTACAATGCTTAAGCATATCTGGAATCTATAGCTACGGCCTGGTCGGCTATATAGTTATTGTGTTTACTAAGAAACGCTTTCGAATTCCTGGCCTATGTAAATTTAGTCATTTGAATGTGTACAACGATGATCATGCATCAACAAAATACAATCAATTGTGCAAACAAAACATTAAACGAAGTGTTAAACGAATTAAATTGATCATGCTATTGACATTGGTATCAATCATAGTCGGAGCTGCAAATCCAACGTATATGTTTTTTAAAACAGGAAAATTTTACAGTTTAACCGGCGTTTTGATACCCGGAATTGATGAAGGTTCACCGAATGAAACATATTTTAATGCAATTTATTTGATCGGCTCATCAATGTATGCCGGCGTTAGTTTGCTTTATGTTCAAATTTGTTCTGGGATTTTGTGCGATACAATTGTGCTGACATCGGATATTATTGTCTTAGAAATGGAAGAACTATCGGAACATTTGGAACGAAATGATTTAAAATCGATCGAAGTACGTTTGCGAGCAAAACGGATCATTTTACAAATATTGAAATCAGATGAATACGTCGCAGACATAAGCGATATTTATTATTGGTATTTTTTTACATCGCCATACCTATTGACATACGCCATTGCTCTAGCCATGTATTCCCAGTATTTGATGGATTTTCCTTGTGGATACGGTTTAGCCGGAATGTCGTATATCCAATTATTTGTTCTGTGCATAATAGGCCAAACCACAGTGGACAGTAGAGAGAAAATTCGGATCGGTCTTGGCAATTTTAAATGGTATTTGATACGTGACATGGAAGTTCGTCGAGATATTGGATTCATTTTGCAACAAGTACAAAATGGCAGTCTTATCACCATGGGACCATTCAATGTATTGAATTATGAATTTGCAGGCATTTTAACCCAACGGATCCACTCGTTCATGATGCTCTTGATAAACTTTGGGAAATAA*GGGCCC*

>MdesOR116 sequence in pcDNA5TO

*GCGGCCGC*CACCATG**GGCAAGCCTATCCCTAATCCTCTGCTGGGCCTGGACAGCACC**GAATTCATGAAAGAACCTCGTCAGATTTGGTCGGATGTGAAAAAAGTGCGAAAAGTATACAATAATTGGGACGCACTCACTCCGGTCAAGCAAATGTATTACCTACATGAAGTGGGCAGATTAAGTGGTATAGTAATTGGAGTACGTACAATGCATGATTGTCGCATCAATGCATCGTCATTTATGATGGCCCCCGTTCTCCTATTGTATTACATTTTGGCAGTTTATACGGTTTACTTTCGTTCAATGAATGGTCAATTTGCGGAAGGACTACAAAGTTTAGCCATATCTGGACTATATTCAAGCGCATTGCTAAGCTATATTTATGTCGTGTTTTCTGAGAAACGCTTTATGTTACACAAATTGAGTAAATTTAGTCAATTAAATGTGTACAACGATGATCATGAACCAACAAAATACAATAAAGTGTGCAAAGAAACCCTTAATAAGAGCGTTAAACGAATTAGATTTATCTTATCTTTGACATATGTTTCAATCATTGTCGGATCTGCTTATCCGACGTATGTTTTTTTTAAAACTGGAAAATTGTACAGTTTAACAGGTGTTTTGATACCCGGATTCGAAGAAGGTTCAAAGGCTGAAAGAAATTGGAATGCAATTTATTTGATCGGTTCATCAATCATTGCCGGCCTTAGTTTGTGTTGTCTTCAAGTTTTTTCTGGGATTGTGTGCGATACGATTATAGTGACATCAGATATAATTGTATTTGAAATTGAACAATTTTCGGAACATTTAGAACGAAATGATTTAAAGCCGAACGAAGTACGTCTACGAGCCAAACGGATCATTTTACAAATATTGAAATCGGATCAATGCACTGCAGAAATAACCGATGGTTTTTATTTTTATTTTTTTTTATCACCATATTTATTGACATACGCGATTGGTGTAGCCATTTATTGCCAGTATTTGATGAATTTTCCTTGTGGATACGGTATAGCCGGAATGTCCTATGTACAATTATTTTATTTGTGCCTAATGGGCCAAATCGTTGTGGACAGTAAAGAGAAAATTCGCATCGCTTTTGGCCTTTTCAATTGGTATTTGATACGTGATATGGAAGTTTTACGAGATATTGGATACATTTTGCATCAAGTACAAAATTGCACCCTTATCACCATGGGACCATTCGAAATATTGAGTTATGAATTTGGAGGCATTTTAACACAACGGATCCTCTCCTTTGTGATGCTTTTGATAAATTTTGGAAAATGA*GGGCCC*

>MdesOR120 sequence in pcDNA5TO

*GCGGCCGC*CACCATG**GGCAAGCCTATCCCTAATCCTCTGCTGGGCCTGGACAGCACC**GATTTTATTAAAAAACCTCGTCAGATTTGGTCGGATGTGAAAAAAGTGCAAAAACTATACAATAATTGGGACGCACTCACTCCGGCCAAGCAAATGCATTGCGTACATGAAGTGGGCAGATTAACTGGTATAATAATTGGAGTGCGTGCGATGCATGATTGTCGCATCGTTGCATCGTCATTTATGATGGCCATCGTTCTCGTATTGTATTACATTTCAGCAGTTTATACAATTTACATTCGTACAATCGATGGACAATTTGCGGAAGGTTTACAATGCTTAAGCATTTCTGGAATCTATTCAAGCGCCATGCTAGGCTATTTATGTTTAGTACTTTTCAAGGAACGCTTTACATTACACAAATTGACTAACTTTAGTCAATTAAATGTGTACATGGATGATCCTGCACCAACAAAATTCAATAAAGTGTGCAAAGGAGCCATTAAACAAAGCGTTCAACGAATTAAATTGATCTTGTTTCTGACATATGTTTCAATCATAGTCGGATCTGCCTATCCGGCATACGTATTTTTTAAAACTGGAAAATTGTACACTTTAACAGGCGTTTTAATACCTGGAGTTTCTGAAAATTCAGCGACTGAAATGTATTTGAATGCAATTTATTTGTTCGTTACATCAATCATTGCCGGCATTAGTTTGCTATTTATTCACTGTATAATTGGAATTTTGTGCGATAATATTGTGGTGACATCG**A**ATATTATTGTCTTAGAAATAGAACAATTATCGGACAATTTGGAACGAAATGATTTAAAGCCGATCGAAATACGTTTGCGAGTCAAACGGATCATTTTACAAATATTAAAATCGGATGAATGTACCGCAGAAACAACCGATGCTTTATATTGGTTTTTTTTCTTATCACCTTATGTATTGACATACGCGATTGGGTTAGCCATGTATTCCCAATATTTGATGGGTTTTCCCTGCGGATACGGTATAGCCGGAATGTCATATGTTCAATTATTTGTTCTGTGCGTAGTAGGCCAGAACACTGTCGACAGTAAAGAAAAAATTCGCATCGCTCTTGGCAATTTCAAATGGTATTTGATACCTGATATGGAAGTTCGTCGAGATATTGGATTCATTTTGCAACAAGTACAAAATTGCTCCGTTTTTACCATGGGACCATTCGATGTATTGAATTATGAATTTGGAGGCATTTTAACTCAACGGATCCTTTCTTTTATGATGCTTTTGATAAACTTCGGGAAATGA*GGGCCC*

Amino acid differences compared to genome: D🡪N

>MdesSNMP1A in pTREX-DEST30

*GCGGCCGC*CACCATGGTATTGCTTAAGTTTCTGAAAACTTTGGATTACGGCAAATTTGCCAATCGATCCGTTATGACGCTATTATTTGGCATTATTGTTCTTGGTGTAATTGTTCCGATGGTTCTCAAATTTGTGGTTAAATCGCAATTGCGTGTAACGCCTGGATCAAAAAACCGTGCACTATTCGAAAGAATTCCTTTTGCTTTGGACCTGGAAATTTATCTCTATAACATATCAAATCCACAAGAAGTTGTTAATGGTGGAAAGCCTAAACTCCACGAAATTGGACCATTTTTCTTTGATGAATACAAACACAAAGAAAATATTATCGATAATGGAATCGATGACACTATCGAATATGATTATGTAAACACATTTATTTATCGTCCGGAAAAAAATGGTCCTGGTTTAACGGGTGACGAAATTGTTACAGTAGCACATCCCATGATGATGCCATTGCTATTAGCTGTAAATATTGAACGTGCCGAACTGTTAGATTTTATAATGGTTGCCATCGATGGCCTTTTTAATAGTCCATCGGACGTATTCTACACTGGTCCAGTTAAAAATTTATTATTCGATGGTATTAAAATTAACTGTTCGAGTGATTCATTTGAAGTGGGCGCAGTATGTTCAGA**A**CTAGACAGCGATGATTATCCGCAAGTGAAGAAAATTAGCGATACAGAATTTACATTTTCTATTTTTGGCAATTC**A**AATGGTACGAGCATTGGACGTTTCCG**G**GCCAAATGCGGAAAAGAAAGTATCAAAGAATTGGGAAAAGTGGTAGCATTCAATGATGAAACACAGTTAGAAGTATGGGACGGTGACGATTGCAATAAAATAATGGGAACCGATGGAACAATTTTTCCACCATTTCAAACCAAAAAACATGATTATTTAATATTTACGCCACAATTATGTCGAACACTTACGGCCAAGCATGTTGGCCAATCCAAATATTCGGGAGTTAAAACACAACGTTTTAAAATTAAAATGGGCATTGAAAATGCAAAAAATCAAACATGTTATTGTCGTGATGTGGATAAATGTCCACCCGAAGGCACATTCGATCTATATCCATGTTCTGGAGTTCCCATTACAGTATCGGCGCCACATTTTTACAATGCTGATCCAGCGATTTTGGAAAAACTGGATGGCCT**G**TCACC**A**AACAAAGA**G**AAACATGC**C**TTCAACTTGGATTTCTATCAATACGCGGGTGCACCAATGTCAGCTCATGGTCGAGTCCAACTCAGTTTCGAGGTTGTGCCAATCGAAAATGTTGAACTGATGAGTAATTTTCAGGAGATGTTTTTACCTTTTCTACGAATCGATGAGGGAACTGATTTGAATAGAAAATTTACGAATATGATAAAATATCAATTATATATAGTTCGGAGCGTCCTGAGATTTTTGAAATATATTTGCATTATTGGCGGATTAGTGGGAATAGTATTGGCATGCGTTGGCCATTTTTTTCAAATGGATGAAGAAAAAGCAGCCAACATTA**T**TGTCGAAGCCGCGTCTGAAAAACAACCGGCCAATCTCAATGGT**GGCAAGCCTATCCCTAATCCTCTGCTGGGCCTGGACAGCACC**TGA*GGGCCC*

Amino acid differences compared to genome: T🡪I

>MdesSNMP1B in pTREX-DEST30

*GCGGCCGC*CACCATGGCCGGACGATTTGACTTTTTGAGACGATTGTCTCCAATAAAATTGGCCATTTTATCAATGATATTATTATT**C**CTTGGTATTGTCTTTTTTGGATTAATTTTTCCCATGATTTTGAAATTTGTCATGAATATGAATTTGCATATTAAACCTGGCGGAAAAAATCGTGCCGTATTTGAGGCTTTACCATTTGTACTAACCATGGATGTATA**C**CTTTTCAATGTGACCAATGTGCATGAAGTGATGGAAGGTGGAAAACCAGAACTTCAACAGATCGGACCCTATCGATTTGACGAATGGAAAACCAAGCAAAATAATATCGATAATGATGAAGATGATACGGTTGAAAGTGATTACATTAACTATTATCACTTTCGGCCCGATCGAACGGACAATGGTTTGACGGGTGAAGAAATAGTTATGGTGCCCCATCCATTGTTATTGTCAATGCTGTTAGCTATAAATTATGAAAAACCGGATATGTTAGATTTTATAACCAAAGCTCTAAATCTTATATTCCAAAATCCGGAGCATTTTTTTTATCATGGGCCAGCTGCTGACCTAATATTTCGAGGAATTCCATTGGATTGTTCAGCAGAAGATTTTGAAGTGTCTGCCGTTTGTTCTGAATTAGAAAGTGATGAATATCC**A**TTGGTCCAAAAATTGAATGACACTTTTATGCAATTTTCGATTATAGGAAATACAAATGCCACCGCAACGGATCATCTAACGGTTAAACGGGGTAAAAAGAATATTATGGATTTAGCCAAAGTGATTGCATCCAACGGAGAGACCGAATTCAGTACATGGCCTGATGATGATTGTAATCAATTTCGTGGCACAGAGGGTACTTTATATCCACCATATTTAAAAAAAACAGACGATTATTGGAT**C**TTTTCGTCATTACTTTGTCGATCATTGCAATTAAAGTACAAAGAACGGACCAAATATAAGGGGATACCGTTGAGTACATATTCATTGGAATGGGGCATTGAAAATACGGAAAAACCATCATGTTATTGCCGTTATGACGATGATGATGAAGATGAAAATGTACCACCAGTTTGCCCATTGAATGGAACTTATGATTTTCAACCATGTTCGGGTGTTCCAATATTGATAACGAGTCCACACTTTTATGGTGCTGATCCACAAATTTTAACCAAATTTAAGTCCGGCATCGAACCAAATG**A**CGAACAACATAGCACCCAATTTGGAATGTATCAATTTGCCGGATTGACGACAACGTTTAATGT**A**AGAGTTCAACTAAGTCTTGAGGTTGTTACGATTGAAGATCATCCGCTATTGGGTAAAGTTCAACCAATGTATTTGCCATTTGCGTGGACATCAAATTCAAATAACCTCAACAAAAAATTCACCAGAATGGTGAAATTTCA**A**TTGGTTTATGCAAAGAGGATGCAAAA**G**GTGATGAAAGTTTTGTGTATTGCATTTGGATTAATGGGTCTTGCGATTTCGGCTACCGTTTACTACCAAAAACTCAGTCTACAGGGAGAATTTGAACGGAATACGAAAGCACCGTCAAACGATATCCAAATACCGGATGCAGCAACAATCGATGCTATCAATAATGTAAAATTAACAAAAGAGAACAATCAGTTCAAGGAA**GGCAAGCCTATCCCTAATCCTCTGCTGGGCCTGGACAGCACC**TGA*GGGCCC*

Amino acid differences compared to genome: G🡪D

>MdesSNMP1C in pTREX-DEST30

*GCGGCCGC*CACCATGAAAATGAATCTGCTCAACACTTTGAAATCGGT**A**GATTTTGTCAAAGTAATGATGATTTCGACATTTTTAATCGCATTTGGATTCATTTTTGGCGGTGTTGTGCTCTCAAAAGTAATTAAAATGGTCATGAAAATGCAACTCCGAATCACACCTGGAACTATGTCAAGGGATATCTACGAA**A**AA**G**TTCCATTCGCTATTGATATGAGATATTTCTTCTTCAATATTACGAATCCGGATGAAGTTATGGCTGGCGGTCAACCACATTTAAATGAGATCGGACCATATTACTTCGAAGAATGGAAAACAAAAGTCGA**T**ATCATTGATGACAATGCCGAAGATACGATGGAATACGATTATATAAATACAATTTATTTTCGGCCGGATTTGAGTGCACCAGGCTTAACTGGTGACGAATTCGTAGTTTTGGCTCATCCGCTAGTATTGGGCATGGCAATGTCAATTAAACGAGAAAGACCTGAATTACTTGAACTCATAAGCAATGCGATAAATGGTCTGTTTCATGATCCAGAGAATATTTTCTTTTCTGGCCGACTGTTTGATCTTTTGTTCGATGGAATTGACTTGGATTGTTCGTCTGAAGCTTTTGAGATTAAGGGAGTTTGCTCGGAACTCGGAAGCGATGACCAGCCCAAAGTCACCGTTATTAATGAAACTACATATTCATTCGCTCTATTCGATCACGGAAACGGTAGTAGCATCGGTCGTTTTAAAGTGATGCGTGGAAAAAAGAACATTAGAGATTTAGGTAAAATTGTTGAGCTTAACGGTGAAACA**G**TACAGGACATTTGGTATGATGATGAATGCAACCAAGTCGATGGAACGGATGGAACAATTTTCCCACCATTTATGAAAAAAGAAGAAGGAGTTCAAGTATTTGTGGCCCAATT**A**TGTCGATCATTCGTGGCTAACTATAAACATCCGTCAAAAGTTTCTGGTATCAAAACAAGCCATTACGAAGTGAATGTTACAGCATCTGCTGATTGTTATTGTAGTGAAGAGGCAAGAC**G**TTGTCCTGCACAAGGACAATTCGATATAATGCCATGTGTTGGAGCCCCAATTACTGTGACTTTGCCCCATTTTCATGAAGCTGATCCTTCTTTGATGGA**T**AATATTAAGGGTGGAATCCATCCCATTCCAGAGAAACATAGTTTCTACTTTGATTTTTATGATTTCGCCGGAGCACCAATCTCTGCGGCTGGACGATTTCAATTAAATTTTGAAGTAGAACCAGTGCAACAAATTGAAATGATGAACAATTTGCAACCCATGCTTTTACCATTCGCATGGTTTGAAAACGGCGTTGATTTAAAAAAGAAATTTGTAAACATGTTAAAATATCAATTGATTCTCGGTCTAAGGTTGAAAAAGATGTTGAAAATTTCTTGCATCATTTTTGGTTTCTTGGGACTAATTGTTGGCTCTACAATGGGGTATTTACAAATGAATGAAGAAACAATGAA**C**AAGACAAAAGCAGTTCAGGTTCAAGCCGCTGCAATTGAAAATG**A**GATATTT**G**ATGATCAAAAAGACGACAATCATGACGAACAAAAA**GGCAAGCCTATCCCTAATCCTCTGCTGGGCCTGGACAGCACC**TAA*GGGCCC*

Amino acid differences compared to genome: Q🡪K; I🡪V; L🡪V; H🡪R; K🡪N; G🡪E; N🡪D

>MdesSNMP1E in pTREX-DEST30

*GCGGCCGC*CACCATGCATTCAATTTTGAGTAAATTGAACACCACGAAAATTATCATTATTTCGTTTTGTTTTCTGATAGCTGGGATATTGCT**G**GGTTTCATTTTAATGCCAATGGGACTAAGAAAATTGATCAAAAGTCAAATTAATTTAAGCCCGAAATCCGACGTGAGAGTTATGTATACAAAGGTTCCATTTCCACTGGAGTTTAAGATCTATCTGTTCAATTTGACAAA**C**AGAGAACATGTTAGA**A**GAGGCGCAAAACCGCATTTGCAGCAAATAGGCCCCTTTTACTTCGAAGAATGGAAAGAAAAGTTC**G**ATCTTTTAGACGATGATACCGAGGATACATTATCGTATCATTACAAAAATACATTCATTTTTCGACCAGATTTGAGTGGACCAGGATTGACAGGAAACGAAATTATCACTATGCCCCATCCATTGATCTCAGGATTGTTGCTTTCGATAAATATTGATAAAAAGCCAATGTTGCCTTTGATTTCCAAAGCTGTAAACATTGTATTCAATGATCCTATCGATATTTTCTGGCAAGGAAGAGTCATGGATATTCTTTTCGATGGAATCCCAGT**C**GATTGCAGTAGTAACGCATTTGAAGCAGCGGCTGTGTGTAGTGTTTTTAGCACAGGTGAAGTGAGCGCAGTAC**T**TCCACTAAATGAAACACATTACAAATTTTCTCTTTTTGGAAGTACAAATGCAACCGATTTGGGAGAATTTAAAGTTATGCG**T**GGCGTGAAGAACTACAATGAAATAGGAAAAGTGGTAGCTTTCAATGGTGAAACGGAAATGGATGTTTGGCCGGA**A**GATGAATGTAATCAATATGTTGGAACTGATTCGACGATATTCAGTTCTCTGATGGATGTAAATGATGGTATTTGGGCTTA**C**GAGCCTGCTATTTGCCGTTCGTTGGGCGCACACTACGTTGGAAAGTCAAAGTATATGGGTGTACCGACGGCTCAATTCGATTTGGACATTGGAAGCGAACAAAATACCAAAGAATGTTTTTGCAGAGATTTTCCAGATGATTGTCCCAAGAAAGGAACATTCGATTTATTCCCTTGCGTTGGTGCACCAATGTTCGGTTCTCT**G**CC**A**CATTTCTTCAATGCCGACCCTTCTTTGTTAGATAATATTGAAGGCCTTTCACCAAATAAAGAGGA**A**CACGCCATATTTATGCATTTTGAAACGTTGTCGGGAACACCAATGTCAGCAGC**T**AAACGGTT**G**CAATTCAACTTGGAAGTTGTTCCAATCGAAGAGGTACCAATTATGCAAGAGATGCGT**G**AAATGCTGTACCCAATGTTTTGGGTTGAAGAAGGTGCCAATTTAAATAAAACTTACGTTAATATGATTAAAAATACTTTGATTCTAGCGGTTCGTATAAAGAATGGGTTCAAATGGATGTGCATCATTGGTGGTATTTGCGGATTAATATTTGCCGGAGTTTTATTTTATATGAAGGAAAATGCTGTTGTTGAAGTTAGTTCGGGTAAGAAGCCCGCATTAAATCGCATCAAAGCC**GGCAAGCCTATCCCTAATCCTCTGCTGGGCCTGGACAGCACC**TGA*GGGCCC*

Amino acid differences compared to genome: G🡪R; N🡪D; R🡪L; Q🡪E

>MdesSNMP1F in pTREX-DEST30 *GCGGCCGC*CACCATGCCCGAATTGGAAATGAAAAGACGCAATTATGGCAAATTGACGCTTATATTTTTGGGCCTTTTTCTAGGTGGCTGCCTAATTTCTTTTTATTTATATTATGTCATATTGGATTTTGAAATTCGAAGGAATATCAGTCTGAGACCGAAGGCAATCGTTCGATGGATTTACATGAATTTACCATTTCCATTAACATTGAAAATCTACATATTTGATGTGTTGAACAAAGAAGAAGTCCAAAATGG**G**GGAAAACCGATCCTGAAACAGATTGG**G**CCCTATGTTTTTCATGAATATGTGATAAAAACGAATACCACTGATTTCAATAGCGAAGATGCACTGGAGTTCATAACTAAAAAAAAATATATATT**C**CGGCCTGACATGAGTCATGGATTAACTGGCGAAGAAATTGTAACGACATTGCATCCGATTATAAATGGCATTGGTTTAGCTGTACAAGCCGAACATAAACAATTCATGCACGTGGCAATAGCTGCAATTAAAGCAATGTTTCACGATCCAATCGATGCATTCTGGACGGGACGTGCAATGGATTTAATATTTGATGGCATCGATATTGATTGTTCGTCGAAAGATCCTTTGGTTCGAATTGCGTGCAAGGAAGTCGATCGGAACGGTGGTGCAACAATTAGACGAGCAAATGAAACAACATTTAAATTTTCAATGCTTGGTGCTAGTAACAATACGGTGAGTGGTGTGTACAAAGTGCTGCGTGGAAAACGATATCCACGTGAATTAGGAAAAATTATTGAACTTGATGGCGAA**G**ATAAATTGAAAGTATGGCGAGGTGAAAAATGTAATGAAATCAAAGGAACAGATGGTTTAATATTTCCACCATTTCAAACGATTGATGAGCCGGTAAACTTTTTTGTGAAACAATTGTGTTTGAACGTTGACCTTCGATATCGTCGAAAAACCTATCTTCGTGGCATTCGAATGCGTTTGTTTGAATATAAAATTGGAAACATTGCCAAAGAGGATCATTTAGAATGTTATTGTCGACATCCGAATCAGTGTCCCATTAATGGTACTATTGATTTGATGCCATGTCTAAAGGTTCC**C**ATTACCGGATCATTGCCCCATTTTTTATATGGCGATCCATCTCTTTTGCAAAATGTGCACGGTTTAAATCCGTCAGAAGTGAATCATTCATTCACCTTAAATATGGAATTGAGAACTGGTGTACCTTTAGCGGGAGCTGGACGATTTCAATTGAATTTTGAACTTGAGCCAGTTCCACAAATCGAAATTATGTCAAATTTACCAAAAATGATTTTTCCATTCATTTGGTTCGAAGAAGGAGCCGATGTTCCCGATGCACTGGTTAATTTGCTAAAATACACTTTAATTTTTGGCACCGGTCTAAATCGTTGTCTGCAATACTTTAGTGTAACTGTTGGACCAATTGGATTCATCATTTGTTTTACGATTTTTTTAATGTTTAAATGTAATATGGTTAGGCTTAAGGGTGCGCCTCGCCGTATTTATGTGAGTGAAAGCCGTGATGTTGAGATTTCAATGATTGGTCACGGTTATCCAAC**G**AGATGGATAAATAATTTGAGAAAAGATGG**G**CGTCCACCAAATTCAACAAAAAGTGCAGGCTAT**GGCAAGCCTATCCCTAATCCTCTGCTGGGCCTGGACAGCACC**TGA*GGGCCC*

Amino acid differences compared to genome: N🡪D
