# Supplementary figures and images for: A Sex Pheromone Receptor in the Hessian Fly Mayetiola destructor (Diptera, Cecidomyiidae)
Source: Front Cell Neurosci. 2016 Sep 7;10:212. doi: 10.3389/fncel.2016.00212 (PMC5013046; doi:10.3389/fncel.2016.00212)

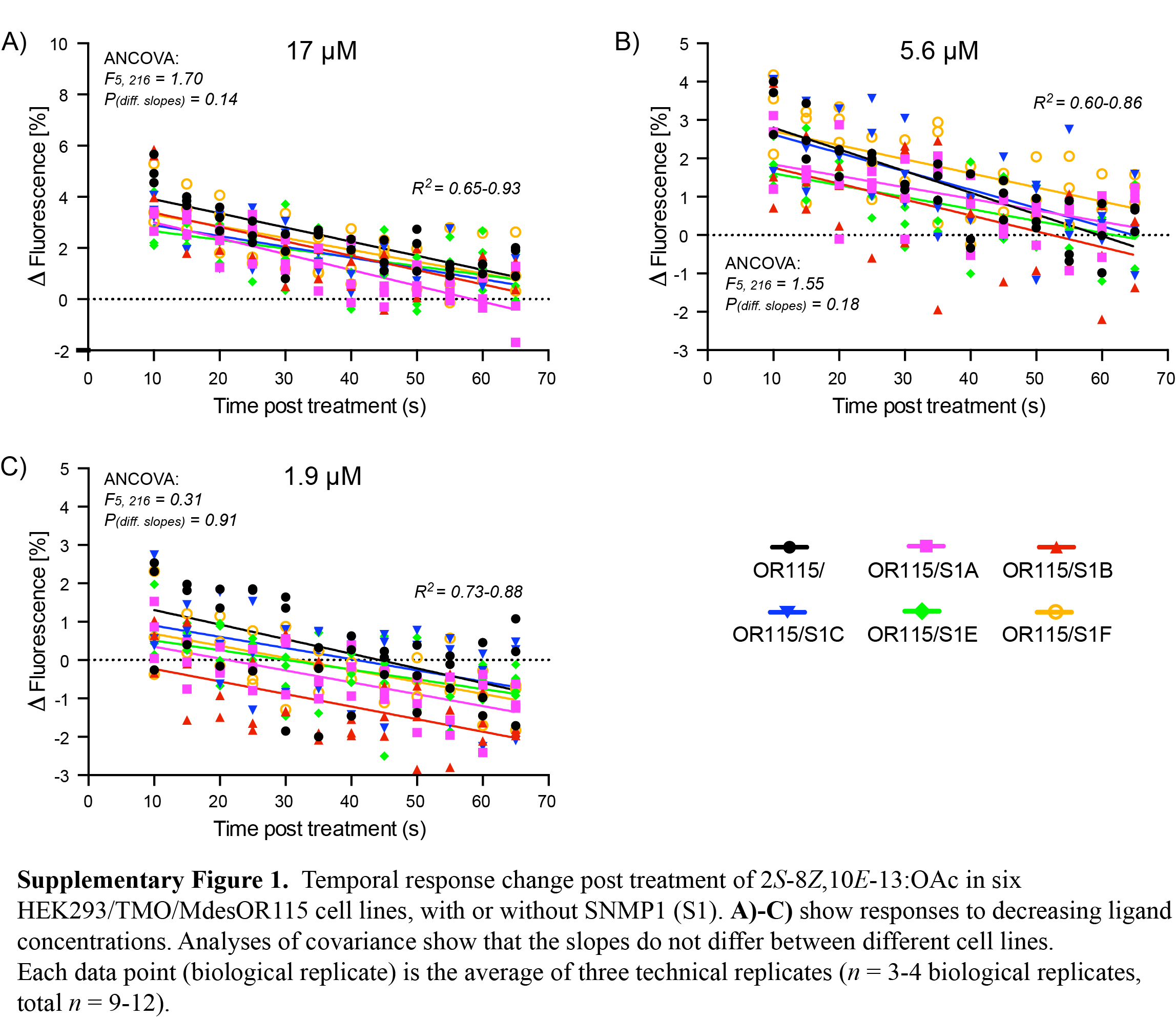

Supplement: Supplementary file 2 [file Image_1.TIF]
